# A Case of Complex Labor, Double Fissure of the Os Uteri, with Cicatrices; Retained Placenta, with Hour-glass Contractions

**Published:** 1862-01

**Authors:** Chas. G. Smith

**Affiliations:** Chicago, Ill.


### ARTICLE II.

### A CASE OF COMPLEX LABOR.

DOUBLE FISSURE OF THE OS UTERI, WITH CICATRICES; RETAINED PLACENTA, WITH HOUR-GLASS CONTRACTIONS.

By CHAS. G. SMITH, M. D., of Chicago, Ill.

Read before the Chicago Medical Society.

On Monday evening, 14th October, I first visited the patient. Having inquired for her previous history, I learned that this was her sixth labor, and that in the previous five, she had never had a living child. The peculiarities of each confinement I could not learn, but only that in the last she had had retained placenta, and crural phlebitis, and was sick a long time. She was now having slight pains, but the membranes had broken, and with every pain came a gush of liquor amnii. On making a vaginal examination, and carrying my fingers as high as possible, I could feel the extremity of a fleshy tumor, in the posterior part of the vagina, but was unable to make out any os uteri. It did not feel like the cord, and there was no pulsation. It did not feel like the placenta, and there was no hæmorrhage. It was too firm for a polypus, and I was in doubt what it was, and waited for time and the progress of the labor to develop its true nature. On making another visit, late that night, Dr. Heydock accompanied me. There was but little change perceptible, still enough to enable us to perceive the extremities of another body, like the one first mentioned, and immediately anterior to it, but not enough to give any accurate idea of what they were.

On Tuesday, the 15th, I visited the woman morning and evening, but the pains were very slight, and accomplished nothing but to irritate and fatigue the patient.

On Wednesday morning, the 16th, the pains started up quite briskly, and it could now be felt that the tumors described, were the anterior and posterior lips of the os uteri, separated by deep fissures on each side, and enlarged by their own growth, or some tumor attached to their inner surface. Dr. Byford saw the patient this noon with me, and detected the head, high above these enlarged growths. As the evening came on, the pains increased more and more, and the internal os could be distinctly made out, with firm cicatrices on each side, and the head pressing down hard on it, without making the slightest dilatation. Late at night, I left the woman quite wearied out, and having occasional and unavailing pains. I gave her some morphine and left directions to be called in the morning, if the pains came on strong and she seemed about to be delivered. I was not summoned, however, till seven o'clock and did not get to the house till half past seven. This was Thursday morning, 17th instant. I found that she had had some few severe pains, and while on her hands and knees, straining a good deal, the child was born, very suddenly and unexpectedly to her attendants, at about six o'clock. It was dead, and gave evidence of having been so for some time. I concluded that the cicatrices alluded to, had, from her great efforts, suddenly ruptured, and, as the vagina was large, allowed the easy passage of the child. On making an examination, I found the pendulous and enlarged lips of the os hanging down the vagina, and that the placenta had not passed from the womb. As it was now, an hour and three quarters, since the birth of the child, I determined to remove it. With my left hand on the fundus, I passed my right easily into the womb, for about half its length, when I found a strong hour-glass contraction, through which a small segment of the placenta was hanging. I may say here, that I did not distinguish any irregularity of surface from the outside, the abdomen of the patient being large and fat and unfavorable to such an exploration. I pulled gently on the placenta, without accomplishing anything, and then passing my hand gradually through the constriction, found that it was adherent to the walls of the womb, for a space of the size of my hand. I then commenced to take it away as well as I could, but it was so firmly attached, that it would not in any sense peel away from the uterus, and it was only by tearing and breaking through its substance, with my fingers, as gently as possible, and as near to the uterine wall, that I was able to get it away at all. The woman was much exhausted by her long labor of nearly sixty hours, and by the manual interference necessary at its close. She rested quietly, though, during the day, and seemed tolerably comfortable at night.

On making my visit Friday morning, the 18th, she seemed to be doing well, with the exception of a slight febrile movement; she said she was getting along very well and felt much better than she expected. In the evening, however, I was summoned to the house, in great haste, with the message, that while a young girl was sitting by her bedside, and waiting on her, she had got out of her bed twice, and run round the room, and was in fact quite crazy. I found her with a pulse of 130, great heat of surface and delirious. The uterus was enlarged, but not very tender on pressure. Dr. BYFORD was called in consultation, and she was directed to take this powder every four hours:

℞—Hydrarg. chlorid. mit., - - - grj.
Opii., - - - - - - - - - - grss.

Alternately, with five drops of the tr. verat. viride every four hours.

The next morning (19th), I stopped the mercurial and opiate, but continued the veratrum viride through the day, till its desired effect on the pulse was produced. The uterus was getting large, and the tenderness increasing, and although the heart's action was lessened by the veratrum, there was no change for the better in the head or general symptoms. She was getting into a typhoid state, from the protracted labor, or the absorption of some poisonous material into the blood, or both. The medicines were stopped, and nothing given but wine and water, and beef-tea, with counter-irritation to the extremities. She lived a day and a half after this, passing from a delirious into a comatose state, in which she died. No post-mortem examination was allowed.

The case seems interesting from the peculiar condition of the os uteri. The tumors spoken of, hung down into the vagina, as much like two tongues, as anything I can describe. On each side above them, in the fissures separating the two, were the firm bands of the cicatrization. It is interesting, also, as proving that adherence of the placenta is the cause nearly, if not quite always, of hour-glass contraction, acting like a splint over the muscles of the womb, to prevent their contraction; and that hour-glass contraction is not readily perceptible from the outside.

